# Associations between dental caries and systemic diseases: a scoping review

**DOI:** 10.1186/s12903-021-01803-w

**Published:** 2021-09-25

**Authors:** Amarpreet Sabharwal, Elizabeth Stellrecht, Frank A. Scannapieco

**Affiliations:** 1 Division of Periodontics, Schulich School of Medicine and Dentistry, DSB 0156A, Western University, 1151 Richmond St., London, ON N6A 5C1 Canada; 2 Department of Periodontics and Endodontics, School of Dental Medicine, University at Buffalo, 3435 Main St., Buffalo, NY 14214 USA; 3 Health Sciences Library University at Buffalo, 3435 Main St., Buffalo, NY 14214 USA; 4 Department of Oral Biology, School of Dental Medicine, University at Buffalo, 3435 Main St, Buffalo, NY 14214 USA

**Keywords:** Dental caries, Oral health, Risk factors, Inflammation, Diabetes mellitus, Type 2, Microbiota

## Abstract

**Background:**

The objective of this study was to evaluate and present evidence from animal and human clinical studies on associations between dental caries and systemic diseases, and to suggest potential mechanisms that might explain such associations.

**Methods:**

An electronic search was conducted of PubMed, Embase and Cochrane Central Register of Controlled Trials for articles published from 2010 to 2020 in the English language. From the initial search, 404 full-text studies were assessed for eligibility. After excluding studies for technical and study limitations, a total of 67 studies were included in the summary tables and additional studies were included in the review to support evidence.

**Results:**

Few systemic disease and conditions were found to be clinically meaningfully associated with caries experience. Best evidence from human and animal studies described association between metabolic diseases and dental caries. Several interesting animal studies were noted that could generate clinical hypotheses and further investigations in rodent models for cardiovascular injury and hyperglycemia. Inadequate data was found to suggest any modifications to current clinical practice or prevention guidelines.

**Conclusions:**

Limited clinical evidence was found connecting several systemic diseases and dental caries. Inadequate data was found to suggest any modifications to current clinical practice or prevention guidelines.

**Clinical significance:**

Understanding of associations between dental caries and systemic diseases play a crucial role in the treatment planning and education of the dental patient.

## Background

Dental caries is one of the most prevalent conditions worldwide [1] and accounts for significant morbidity [2]. Importantly, the prevalence of untreated dental caries has increased [1, 2]. While there is a direct effect of untreated dental caries on oral health and associated quality of life, identification of indirect associations between dental caries (including untreated dental caries) and systemic health are of potential interest but have received little attention [3].

Associations have been more studied between periodontitis and systemic diseases and the contribution of oral inflammation and microbiota to diseases such as atherosclerosis, diabetes mellitus, pneumonia, chronic obstructive pulmonary disease, rheumatoid arthritis (RA) and Alzheimer disease (AD) [4–6]. In addition to epidemiologic evidence, laboratory and animal studies provide biological plausibility for periodontal-systemic associations [7, 8].

While both dental caries and periodontitis are biofilm-mediated diseases, the pathogenesis of dental caries is complex and multifactorial and differs from periodontal disease. Dental caries is a biofilm-mediated disease with multiple contributing factors that drives net localized demineralization of the teeth [9]. The plausibility of systemic consequences from untreated dental caries and mechanistic role of the associated oral microbial-inflammatory process in these associations requires further inquiry through human and animal studies. The ability of oral microbiome to spread into systemic circulation from dental caries is plausible and would parallel mechanisms already studied for periodontal disease. In dental caries, involvement of root canal space or marginal periodontium are the most likely pathways for direct systemic extension of oral microbiota [10]. Host factors and pathogenic traits in oral microbiota can promote dental caries and increase the likelihood of oral-systemic spread. Such factors would include diseases [11] and medications [12] that result in reduced saliva production, adhesin expression in S.mutans for collagen binding [13–15], dysbiosis of the oral microbiota [16, 17], genetic factors that predispose to dental caries and share common mechanistic underpinnings with systemic diseases [18].

The hypothesis of systemic spread of oral microbiota from carious lesions is reasonable but mechanisms by which systemic diseases exacerbate dental caries requires considerable future research. Metabolic diseases such as diabetes and obesity share various common environmental determinants with dental caries, including hyperglycemic state and high-carbohydrate/sugar-rich diet [19]. Our current understanding of metabolic disease-dental caries associations and use of animal models [20–27] can serve to expand understanding of associations between dental caries and other systemic diseases. Animal models allow for study of systemic variables in dental caries due to the ability to longitudinally study disease phenotype within a reasonably short time frame.

This scoping review compiled and evaluated recent evidence from animal and clinical human studies that assessed associations between dental caries and systemic diseases and potential mechanisms for such associations. Specifically, a scoping review was undertaken to establish areas in which evidence on associations between dental caries and systemic diseases is available [28].

## Methods

### Data sources

An electronic search was conducted by a health sciences librarian (ES) in June 2021 in PubMed, Embase and Cochrane Central Register for Controlled Trials. Results were limited to articles published from 2010 to 2020 in the English language.

### Search strategy

The following search strategy in PubMed utilized both keyword terms in the title and abstract fields as well as Medical Subject Headings (MeSH) to identify possible qualifying articles: (((((“dental caries“[MeSH Terms]) OR caries[Title/Abstract]) OR carious lesions[Title/Abstract]) OR carious lesion[Title/Abstract])) AND (((((((((((((((((((((((((((((((((((((((“neoplasms“[MeSH Terms]) OR cancer[Title/Abstract]) OR metabolic syndrome[Title/Abstract]) OR “metabolic syndrome“[MeSH Terms]) OR obesity[Title/Abstract]) OR “obesity“[MeSH Terms]) OR cardiovascular diseases[Title/Abstract]) OR cardiovascular disease[Title/Abstract]) OR “cardiovascular diseases“[MeSH Terms]) OR myocardial infarction[Title/Abstract]) OR heart disease[Title/Abstract]) OR heart diseases[Title/Abstract]) OR diabetes[Title/Abstract]) OR “diabetes mellitus“[MeSH Terms]) OR atherosclerosis[Title/Abstract]) OR cerebrovascular disease[Title/Abstract]) OR cerebrovascular diseases[Title/Abstract]) OR “cerebrovascular disorders“[MeSH Terms]) OR asthma[Title/Abstract]) OR “asthma“[MeSH Terms]) OR pneumonia[Title/Abstract]) OR “pneumonia“[MeSH Terms]) OR chronic obstructive pulmonary disease[Title/Abstract]) OR “pulmonary disease, chronic obstructive“[MeSH Terms]) OR allergies[Title/Abstract]) OR “hypersensitivity“[MeSH Terms]) OR “respiratory tract diseases“[MeSH Terms]) OR arthritis[Title/Abstract]) OR “arthritis, rheumatoid“[MeSH Terms]) OR Alzheimer Disease[Title/Abstract]) OR “Alzheimer’s Disease” [MeSH Terms]) OR dementia[Title/Abstract]) OR “dementia“[MeSH Terms]) OR “inflammatory bowel diseases“[MeSH Terms]) OR crohn disease[Title/Abstract]) OR “osteoporosis“[MeSH Terms]) OR osteoporosis[Title/Abstract]) OR “joint diseases“[MeSH Terms]) OR systemic[Title/Abstract]).

This search was translated and updated for Embase and Cochrane Central Register of Controlled Trials accordingly.

### Data filtering

The results obtained using search strategy described above were deduplicated and further managed in an online workflow management system for scientific reviews (https://www.covidence.org/). After removal of duplicates, titles were examined by one author (AS) and articles unrelated to dental caries were removed. For retained articles, after title-based filtering, their eligibility was assessed by abstract-based filtering by two authors (AS and FAS). If articles were considered unrelated to scope of this review using criteria identified below, they were excluded. Articles were removed for various technical reasons such as wrong outcome measures, inadequate statistical information, narrow participant enrollment such as studies with one gender enrollment. Articles that were published in potentially predatory journals were also removed if the corresponding journal was listed in Beall’s list (https://beallslist.net/) and was not listed in the Directory of Open Access Journals (https://doaj.org/). When articles were on the topic of associations between dental caries and systemic diseases, systematic and retrospective reviews and data analysis of health records were excluded. Studies were also excluded if dental caries was not the primary variable and was studied as a subset of oral health and/or systemic disease was studied as a subset of overall health. In addition, infective endocarditis was removed from our search criteria as it has been extensively reviewed previously.

Clinical human and animal studies were included where associations between dental caries and a systemic disease were explored or a potential mechanism was elucidated and they did not meet any of the aforementioned exclusion criteria. Using the filtering criteria above and after full-text screening by two authors (AS and FAS) studies were included in the summary tables and additional studies were included in the review to support evidence. If one author agreed to inclusion after full-text screening, the corresponding article was included.

Individual studies were tabulated and brief description of the following parameters were provided: name of first author, year of publication, number of participants, country of study participants, study groups (treatment and control), study population (human or animal), objective of the study, study design, outcomes including statistical parameters and conclusions (Tables 1, 2, 3, 4, 5 and 6).

## Results

After deduplication, the initial search yielded 4817 results. 404 full-text articles were assessed for eligibility and further 133 studies were excluded for various technical reasons described above. The remaining 271 full-text articles were assessed, and after excluding studies where dental caries and/or systemic disease was not the primary variable of interest, and excluding literature reviews and data analysis of health records, 67 studies were included in the summary tables.

Studies were included on the following systemic diseases: coronary artery disease (Table 1), congenital heart disease (Table 1), peripheral artery disease (Table 1), hypertension (Table 1), diabetes (type I and II) (Table 2), obesity (Table 2), metabolic syndrome (Table 2), cystic fibrosis (Table 3), asthma (Table 3), ulcerative colitis (Table 4), Crohn’s disease (Table 4), cerebral palsy (Table 5), attention deficit hyperactivity disorder (Table 5), rheumatoid arthritis (Table 6), systemic lupus erythematosus (Table 6) and chronic kidney disease (Table 6). A total of 56 human studies and 11 animal studies were included in the summary tables. Relatively, more studies on metabolic diseases (type I diabetes, type II diabetes, obesity and metabolic syndrome) were included in this review (40 total, 32 human and 8 animal) when compared to evidence found in other disease groups. Within 56 human studies included, 29 studies were case-control, 17 were cross sectional and 10 were longitudinal studies.

**Table 1:** Evidence on cardiovascular diseases and caries

Study	Objectives and study design	Study type	Number of participants	Location of study	Outcomes and conclusions
Human studies on cardiovascular diseases and caries					
Coronary artery disease (CAD)					
Fadel et al. [29]	Evaluate caries (using Cariogram) and periodontal disease risk in patients with CAD	Case-control	127 (54 cases, 73 controls)	Saudi Arabia	Outcomes: For lifestyle factors, patients with CAD consumed significantly less sugar, used less fluoride toothpaste and had worse periodontal health (*p* < 0.005). Non-significant differences were noted between groups for caries [mean decayed, missing, and filled surfaces (DMFS)] but both groups had relatively higher caries risk (assessed by Cariogram); gingival recession was correlated positively with onset of CAD Conclusion: Long-term studies are needed to validate the use of Cariogram in patients with CAD
Congenital heart disease					
Siahi-Benlarbi et al [30]	Investigate oral and intestinal C*andida* colonization and dental caries status [decayed, missing, and filled teeth (DMFT/dmft)] in immunocompromised pediatric cohort (2–16 yrs) by comparing patients with heart transplants (n = 31) and congenital heart disease (n = 24) to controls (n = 23)	Case-control	78 (55 cases, 23 controls)	Germany	Outcomes: DMFT/dmft (*p* < 0.001) and intestinal candida colonization (*p* = 0.027) was significantly lower in transplant patients compared to other groups; significant correlation (*p* < 0.001) was noted between incidence of *Candida* and dental caries; significant difference *p* < 0.001) was noted in between groups for positive serum concentration of *Candida*-mannan antigen with no *Candida*-anti-mannan antibodies in the heart transplant group Conclusion: There is a correlation between oral *Candida* colonization and dental caries
Peripheral arterial disease					
Soto-Barreras et al [31]	Compare caries (DMFT), periodontal (probing depth, attachment loss), microbiological (subgingival flora) and biochemical [C-reactive protein (CRP)] markers between patients with and without peripheral arterial disease (measured by ankle-brachial index)	Case-control	60 (30 cases and controls)	Mexico	Outcomes: Prevalence of periodontitis (*p* = 0.03) and missing component (*p* = 0.04) of DMFT were significantly higher in the peripheral arterial disease group; levels of CRP were significantly higher in the peripheral arterial disease group (*p* = 0.04); *P. gingivalis* was non-significantly higher in peripheral arterial disease group and *S. mutans* did not show statistical difference between groups; after controlling for risk factors and confounders, periodontitis was positively associated with peripheral arterial disease Conclusion: There was positive relationship between periodontitis and peripheral arterial disease [Odds Ratio (OR = 8.18)]
Hypertension					
Ostalska-Nowicka et al. [32]	Evaluate association between dental caries and hypertension in children and adolescents aged 6–18 years of age. Blood pressure, dental caries (dmft/DMFT), sIgA and various serum biochemical parameters were recorded. These parameters were compared with normotensive control group	Case-control	109 (65 cases, 44 controls)	Poland	Outcomes: Significantly higher uric acid concentration was noted in hypertensive group when compared to normotensive controls (*p* = 0.047). Salivary cortisol and α-amylase levels were significantly higher in hypertensive group (*p* = 0.002 and *p* = 0.004 respectively). Multivariate analysis showed dental caries was associated with hypertension (*p* < 0.0001) Conclusion: Dental caries in children and adolescents may be considered as a contributory factor to hypertension when other causes have been ruled out
Animal study on cardiovascular diseases and caries					
Coronary artery disease					
Kesavalu et al [33]	Animal study to investigate the effect of *S. mutans*, OMZ175 in atherogenesis using an apolipoprotein E deficient (ApoE^null^) mouse model by comparing groups with and without balloon angioplasty injury and appropriate controls				Outcomes: Histomorphometric analysis of aortic sections in angioplasty with *S. mutans* group showed significant increase (*p* < 0.05) in plaque area and intimal/medial thickness when compared to controls; immunohistochemical studies showed significantly increased (*p* < 0.05) macrophage invasion in the adventitia and upregulation of Toll-like receptor (TLR) 4 in angioplasty with *S. mutans* group when compared to controls Conclusion: *S. mutans* accelerated atherosclerotic plaque growth, macrophage invasion and TLR4 expression after aortic injury

**Table 2:** Evidence on metabolic diseases and caries

Study	Objectives and study design	Study type	Number of participants	Location of study	Outcomes and conclusions
Human studies on metabolic diseases and caries					
Diabetes					
Hegde et al. [34]	Evaluate salivary composition in caries-active (>10 decayed teeth) diabetic patients when compared to caries-active (>10 decayed teeth) controls	Case-control	120 (60 cases and controls)	India	Outcomes: Salivary alkaline phosphatase was significantly higher in diabetic caries-active patients when compared to controls (*p* < 0.001) and salivary calcium ions were significantly higher in non-diabetic caries-active patients (controls) when compared to diabetic caries-active individuals (*p* < 0.001) Conclusion: Salivary composition with regards to calcium and alkaline phosphatase is significantly different between caries-active diabetic and non-diabetic individuals
Lai et al. [35]	Evaluate caries prevalence [International Caries Detection and Assessment System (ICDAS)], dietary and oral hygiene variables, diabetic control (HbA1c), oral microbiota (DNA-DNA) and plaque acidogenicity by comparing non-diabetic and diabetic (good control with HbA1c ≤ 7.5; poor control with HbA1c ≥ 7.5) pediatric cohorts.	Case-control	204 (68 cases, 136 controls)	Italy	Outcomes: Consumption of sugary beverages and snacks were significantly higher in diabetic group compared to non-diabetic group (*p* = 0.03 and *p* = 0.04 respectively) and similarly, diabetics with poor control consumed significantly higher sugary beverages and snacks compared to diabetics with good control (p>0.01 and *p* = 0.03 respectively); significantly higher caries free participants in diabetics with good control when compared to diabetics with poor control (*p* < 0.01); significant difference in use of fluoridated toothpaste and tooth brushing ≥ 2 mins were noted between the diabetic subgroups (*p* = 0.03 each); significant differences were noted between diabetic subgroups for all primary cariogenic bacteria except ‘other mutans streptococci’; pH values of plaque were significantly different between both groups and diabetic subgroups (*p* < 0.01 each) Conclusion: Diabetic children with good control may be considered low caries risk while diabetic children with poor control may be considered high caries risk.
Majbauddin et al. [36]	Investigate association between dental caries (DMFT) and HbA1c [controlled diabetes mellitus type II (T2DM) with HbA1c ≤ 7; and uncontrolled T2DM ≥ 7]	Cross sectional	91 (46 controlled and 45 uncontrolled diabetics)	Philippines	Outcomes: Significantly worse oral hygiene parameters including frequency of tooth brushing (*p* = 0.04), frequency of flossing (*p* = 0.002), lack of oral health education (*p* < 0.001) and irregular dental visits (*p* < 0.001) were noted in uncontrolled T2DM groups; significantly higher decayed teeth (DT) (*p* = 0.04), MT (*p* = 0.002) and DMFT(*p* < 0.001) were noted in uncontrolled T2DM group; absolute value of DT was significantly correlated with serum HbA1c levels (*p* = 0.005) and education level (*p* = 0.04) and significant correlation of DMFT index were noted between serum HbA1c and regular dental visits (*p* < 0.001) and receiving oral health education (*p* = 0.004) Conclusion: There is association between markers of dental caries and serum HbA1c levels
Singh-Hüsgen et al. [37]	Evaluate caries (DMFS/dmfs) prevalence, periodontal parameters (Silness & Löe and papillary bleeding indexes) and subgingival microbiota in diabetic [diabetes mellitus type 1 (T1DM)] and phenylketonuria pediatric cohorts by comparing it with a control group	Case-control	238 (138 cases, 100 controls)	Germany	Outcomes: Statistically significant difference was noted for caries prevalence in primary dentition between the three groups but no statistically significant difference was noted in permanent dentition; Silness and Löe index was statistically significantly higher in diabetic group compared the other two groups; papillary bleeding index was significantly higher in diabetic group compared to controls; statistically significant differences were noted for *Lactobacillus sp., Lactobacillus casei *and *Porphyromonas gingivalis *between groups Conclusion: Children with phenylketonuria demonstrated a higher caries experience in their primary dentition and diabetic children showed a slightly higher risk of developing periodontal disease
Al-Badr et al. [38]	Compare prevalence of dental caries between 6-12-year-old type 1 diabetic children and controls matched by age and gender. Diabetic and non-diabetic participants were compared for oral hygiene, socioeconomic status, caries burden (DFT/dft), salivary microbiota and salivary pH	Case-control	173 (69 cases, 104 controls)	Saudi Arabia	Outcomes: Mean DFT/dft scores between groups were non-significantly different between groups. Diabetic children showed significantly higher dentist visits (p = 0.04) and lower consumption of sugary foods (p = 0.003). Further, diabetic children had significantly lower salivary pH (p = 0.01) and higher *Lactobacillus *counts (p = 0.04) when compared to non-diabetics Conclusion: There was non-significant difference in caries burden between type 1 diabetic and non-diabetic children. The reduced salivary pH and higher *Lactobacillus *counts may indicate a higher risk in type 1 diabetic children.
Kamran et al. [39]	Compare dental caries burden (DMFT) in type 1 diabetic children and healthy controls. Participants were recruited, ranging in age from 9-14 years. Additionally, oral hygiene habits, duration of diabetes and HbA1c levels were recorded	Case-control	200 (100 cases and controls)	Iran	Outcomes: Mean DMFT in diabetic and control groups were 2.60 ± 1.25 and 2.52 ± 1.26 respectively. No significant difference between groups was noted for DMFT (p = 0.654). For oral hygiene, participants who flossed regularly showed significantly lower DMFT when compared to individuals who did not use dental floss (p = 0.001). No significant difference was found in mean DMFT due to diabetes duration or HbA1c levels. Conclusion: Type 1 diabetes alone may not affect dental caries burden, but oral hygiene is important in controlling dental caries
Pachonski et al. [40]	Compare caries burden (DMFT) in type 1 diabetic participants (10-18 years) when compared to controls. Cases were divided into poorly (PC) and well controlled (WC) sub-groups based on glycemic control (HbA1c of 7.5% cutoff).	Case-control	75 ( 50 cases, 25 controls)	Poland	Outcomes: Statistically significant difference in DMFT was noted between PC and WC subgroups (p = 0.04) with PC subgroup showing highest mean DMFT values (5.8 ± 3.75). No other statistically significant differences were noted. Conclusion: Type 1 diabetic patients may show significantly higher caries burden.
Schmolinsky et al. [41]	Assess effects of type 2 diabetes and metabolic control on coronal caries. This study was a 11-year follow up. DMFS, HbA1c, behavioral, socioeconomic, education level, smoking status and dental home care data was recorded. Parameters from poorly controlled, well controlled diabetes and non-diabetic controls were compared	Longitudinal	2028	Germany	Outcomes: For dental caries, progression of DMFS rates were significantly higher in poorly controlled diabetics compared to other groups (p = 0.01). Importantly, rate of DMFS change differed significantly when duration of diabetes was ≥ 5 years and non-significantly different for duration of diabetes ≤ 5 years. Rate of change of HbA1c levels increased proportional to DMFS index (B=0.046, linear effects model) Conclusion: Participants with poorly controlled diabetes and longer duration of disease (≥ 5 years) are at increased risk for caries progression (ΔDMFS)
Obesity					
Alm et al. [42]	Investigate association between body weight [body mass index (BMI)] and caries prevalence [decayed, extracted, and filled surfaces(defs)/ decayed and/or filled approximal surfaces (DFS_a_)] followed from pre-school years to young adulthood at 3, 6, 15 and 20 years of age	Longitudinal	402	Sweden	Outcomes: At 3 years of age, no association was noted between overweight/obesity and caries; at 6 years of age, significantly higher caries prevalence was noted in obese children when compared to normal weight children (*p* = 0.04) with a OR=2.5 times that of normal weight children; at both 15 and 20 years of age, overweight/obese children had significantly higher caries prevalence when compared to normal weight young adults (*p* < 0.05) Conclusion: Overweight and obese adolescents and young adults had significantly more caries than normal-weight individuals. Emphasis should be placed on need for preventive approaches that address lifestyle factors that affect obesity and dental caries
Basha et al. [43]	Assess association between obesity (BMI) and dental caries (DMFS/DMFT) prevalence and increments in 13 year-old adolescents with 3 years follow up	Longitudinal	764	India	Outcomes: Significantly higher number of girls were overweight/obese compared to boys (*p* = 0.04) and prevalence of dental caries was significantly more in boys compared to girls (*p* = 0.04 ); after 3 years, significantly more adolescents had dental caries compared to baseline (*p* = 0.001); mean caries scores were higher in obese and overweight children compared to normal weight children at both examinations-baseline and 3 years (*p* < 0.05); children with obesity and overweight status had a 3.7 times greater chance of developing caries after adjusting for confounders Conclusion: Obese and overweight adolescents were at a higher risk of developing new caries in a 3 year follow up period
Chala et al. [44]	Evaluate non-linear associations between BMI and dental caries (untreated dental decay). BMI was treated as continuous variable and a multivariable Poisson regression model was established	Cross sectional	101	Morocco	Outcomes: Adjusted multivariate analysis revealed that age at beginning tooth brushing and BMI, both below and above the normal range were associated with increase of number of dental caries. A significant quadratic effect between BMI and the rate of untreated dental decay was noted (p value for non-linearity was <0.001 and for overall effect was <0.001) Conclusion: A U-shaped trend in the association between dental decay and BMI was found which means an increased rate of untreated dental decay was associated with both under- and over-weight status
Costacurta et al [45]	Evaluate the association between obesity (BMI, body fat mass and body fat free mass) and dental caries (DMFT/dmft) and the impact of lifestyle, dietary and oral hygiene parameters on dental caries in obese pediatric patients by comparing four groups-normal weight with and without caries and pre-obese-obese with and without caries	Cross sectional	96	Italy	Outcomes: Pre-obese-obese children had higher DMFT (*p* = 0.04) and dmft (*p* = 0.03) indexes compared to normal weight participants; significant correlation was noted between dmft/DMFT and body fat mass (*p* = 0.03/0.02 respectively); for lifestyle and diet, there were significant differences between groups for intake of sugary drinks (*p* = 0.005), frequency of sugar intake limited to main meals (*p* < 0.001) and sedentary lifestyle (*p* = 0.01) with higher percentage of participants in the preobese-obese group with caries Conclusion: There is a direct association between dental caries and obesity and specific dietary habits may be considered risk factors that are common to both dental caries and childhood obesity
Goodson et al. [46]	Evaluate association of childhood obesity (BMI) and dental caries [DT and filled teeth (FT)] by comparing obese, overweight, normal weight and underweight children	Cross sectional	8275	Kuwait	Outcomes: The percentage of DT and FT varied inversely to body weight and the differences between groups for DT and FT were statistically significant; reduced prevalence of dental decay in obese children was significant for both primary and permanent dentition but was comparably less in permanent teeth than in primary teeth Conclusion: An inverse relationship between obesity and dental caries argues against the hypothesis that sugar is necessary and sufficient for dental decay and is a leading co-factor in obesity. The reasons for the inverse relationship noted in this study are not entirely clear
Hall-Sculin et al. [47]	Evaluate association between caries (DMFT) in late childhood (7-9 years) and obesity (BMI) in adolescence (12-16 years) and define strategy for prevention	Longitudinal	2953	England	Outcomes: BMI categories in adolescence were not significantly associated with presence of caries in late childhood (*p* = 0.6) or adolescence (*p* = 0.06); obesity was not significantly associated with gender (*p* = 0.9); statistically significant association was not seen between BMI and ethnicity (*p* = 0.02) Conclusion: Caries in late childhood was not shown to be associated with obesity in adolescence and no association was noted between obesity and diabetes in adolescence.
Li et al. [48]	Evaluate associations between obesity (BMI, waist circumference, waist-height ratio, waist-hip ratio) and dental caries (DMFT) in adolescents (12 years) with 3 and 6 year follow ups	Longitudinal	282	China (Hong Kong)	Outcomes: Significant increase in percentage of underweight adolescents were noted during the period of observation (*p* < 0.001); BMI, waist circumference, waist-hip ratio and waist-height ratio were associated with frequency of tooth brushing at 3 and 6 years follow ups; prevalence of dental caries increased with increase in duration of follow up (25.5% at baseline to 62.1% at 18 years of age); at 6 years follow up, mean DMFT score of participants with waist-hip ratio below median was significantly lower than mean DMFT score of participants with waist-hip ratio above median at previous follow up (*p* = 0.03) Conclusion: Longitudinal association was noted between central obesity and dental caries among adolescents between 15 and 18 years of age
Modéer et al. [49]	Evaluate if childhood obesity (BMI-adjusted for age and gender) is associated with reduced stimulated salivary flow rate and dental caries (DMFT/DMFS) by comparing obese and normal weight (control) groups	Case-control	130 (65 cases and controls)	Sweden	Outcomes: Obese participants showed significantly higher number of decayed surfaces (*p* = 0.008) and significantly lower flow rate of stimulated whole saliva (*p* < 0.001) compared to controls; obesity (BMI-adjusted for age and gender) as a continuous variable was significantly associated with decayed surfaces (OR=1.3) Conclusion: Childhood obesity is associated with reduced stimulated whole saliva flow rate and dental caries.
Peng et al. [50]	Evaluate association between adiposity (general, central and peripheral) and dental caries (DMFT and significant caries index-SiC) in early adolescence. Metrics of adiposity used were BMI, waist and hip circumferences, triceps skinfold thickness, waist-height and waist-hip ratios	Cross sectional	514	China (Hong Kong)	Outcomes: Gender was associated with adiposity with boys having significantly higher waist-height ratio (*p* < 0.01), BMI (*p* < 0.05), waist circumference (*p* < 0.001) and waist-hip ratio (*p* < 0.001) than girls; children brushing less than once daily had significantly higher BMI, waist circumference and waist-hip ratio than those brushing at least once daily (*p* < 0.05); parental education was associated with prevalence of dental caries (*p* < 0.01), SiC index prevalence (*p* < 0.05) and mean DMFT (*p* < 0.01); dental caries experience was associated with adiposity and there was a significant correlation between DMFT and waist-hip ratio (*p* = 0.03); regression models identified that dental caries was associated with adiposity Conclusion: Dental caries experience was associated with central and peripheral adiposity but not general adiposity
Sánchez-Pérez et al. [51]	Evaluate effect of BMI on tooth eruption in a pediatric cohort (n=88) by studying dental caries (DMFT/dmft and DMFS/dmfs), BMI and tooth eruption timings	Longitudinal	88	Mexico	Outcomes: Significant increase in children with 85^th^ percentile of BMI over the follow up period (*p* < 0.001); significant association was noted between number of erupted teeth and BMI (*p* < 0.001) and longitudinal effect estimated by mixed model indicated higher eruption rate with increase in BMI over time; mixed model fitted for caries (dmfs) showed that children with high BMI had significantly lower levels of dental caries (*p* < 0.01) and participants from lower socioeconomic resources had significantly higher dmfs scores (*p* = 0.01) Conclusion: Children who were overweight had increased eruption rate and lower caries index.
Akarsu et al. [52]	Evaluate association between BMI and dental caries in 20-30-year-old adults without any chronic diseases. 394 participants were divided into groups based on BMI (normal weight, overweight and obese) and compared	Cross sectional	394	Turkey	Outcomes: Mean DMFT was statistically significantly higher in obese group when compared to normal and overweight groups (p = 0.001 each). No statistically significant difference was noted between mean DMFT of normal and overweight groups (p > 0.05) Conclusion: A positive relationship was noted between obesity and higher DMFT index.
Fraiz et al. [53]	Determine association between overweight/obesity (excess body weight, measured as BMI) and prevalence of dental caries (dmft) in a cohort of 4-5-year-old school children. In addition, information on SES, schooling of parent/caregiver and snack consumption limits were collected	Cross sectional	686	Brazil	Outcomes: 16.6% were overweight and 10.9% were obese. A multivariate model showed household income per capita [PR=0.804 (0.665-0.972)], age of the child [PR=2.025 (1.001-1.029)]and snack consumption limit [PR=0.839 (0.732-0.962)] were associated with greater prevalence of dental caries. Conclusion: Excess body weight was not associated with dental caries. Dental caries was significantly higher in older preschoolers, participants with lower household income and in households where parents had lower limit on snack consumption.
Frias-Bulhosa et al. [54]	Determine associations between BMI and dental caries (DMFT) in 13-year-old participants.	Cross sectional	181	Portugal	Outcomes: No significant difference was found for dental caries (DMFT) between groups by BMI (underweight, normal, overweight, and obese). However, frequency of oral hygiene was significantly associated with DMFT ≤ 6 (p = 0.041). For severe dental caries (DMFT > 6), no oral hygiene at night was a significant risk factor (p = 0.006) Conclusion: No significant association was found between BMI and dental caries in cohort of 13-year-old participants.
Guare et al. [55]	Compare caries [dentin (DC) and enamel (EC)] and caries risk between normal weight (NW) and overweight/obese (OW) 6-12-year-old children. BMI, caries using ICDAS system (two categories: EC/DC and DC) and caries risk using the caries management by risk assessment (CAMBRA) system were recorded and analyzed by logistic regression.	Case-control	91 (41 cases, 50 controls)	Brazil	Outcomes: Caries burden was similar in both groups for EC/DC threshold but higher in NW group for DC threshold (p = .009). Further, caries risk classification was similar to both groups and logistic regression analysis showed that OW group was less likely to demonstrate proximal caries (OR=0.33), thick biofilm (OR=0.36) and have high (OR=0.367)/moderate-high (OR=0.19) caries risk Conclusion: Children in the OW group had lower caries experience and risk compared to NW children.
Karki et al. [56]	Evaluate associations of untreated dental caries [grade of severity of untreated dental caries (GUDC)] in groups based on BMI and stratified based on three systems [WHO, International Obesity Task Force (IOTF), Nepalese growth reference] in WHO index age groups of 5-6, 12 and 15-year-old school children. Additionally, demographic information, oral hygiene and food consumption habits were noted	Cross sectional	1135	Nepal	Outcomes: Untreated dental caries was common in youngest age group (5-6-year-old) (*p* < 0.001), in participants with infrequent tooth brushing (p = 0.007), and frequent consumption of sugary foods (p = 0.014). BMI (low or high) was associated with severity of untreated dental caries (GUDC) [for low BMI, RR=1.09; for high BMI, RR=1.07] Conclusion: Children with high or low BMI may be at risk for dental caries due to shared common risk factors, prominently dietary factors.
Kennedy et al. [57]	Evaluate associations between BMI and severe early childhood caries (S-ECC) (dmfs) in children under 6 years of age.	Longitudinal	150	Canada	Outcomes: Multiple linear regression analyses showed no significant relationship between dmfs and BMI z-scores. However, a significant relationship between BMI z-scores and family income (< $28,000/year), registered first nation status, reporting of inadequate physical activity (p = .008, .005 and .02 respectively) Conclusion: No significant relationship between BMI and S-ECC was noted but socioeconomic status was an important confounding variable.
Lock et al. [58]	Evaluate association between obesity (BMI) and change in dental caries (ΔDMFS) in 12-year-old schoolchildren. This study was a 2.5-year follow-up of a cross-sectional study (baseline) and 801 participants were followed up	Longitudinal	801	Brazil	Outcomes: DMFS increased by 0.86 (0.65-1.07), 0.91 (0.59-1.23) and 0.42 (0.03-0.80) for normal, overweight, and obese groups respectively. Further, obese group had significantly lower ΔDMFS compared to normal weight group (*p* < 0.05). While no significant association was noted between BMI categories (normal, overweight, and obese) and ΔDMFS, a polynomial model showed inverse relationship between increasing BMI and decreasing ΔDMFS (p < 0.05). Conclusion: An inverse association was seen between obesity and Δ DMFS in this longitudinal follow-up.
Serrano-Pina et al. [59]	Determine associations between obesity (BMI) and dental caries [DMFT/deft and TD (total decay)] in 8-12-year-old schoolchildren.	Cross sectional	331	Mexico	Outcomes: Dental caries prevalence was 32.4% (29.7-35.2) and mean DMFT was 0.64 ± 1.00. Further, statistically significant negative correlation was noted between BMI and TD (r = -0.127, p = 0.021); BMI and deft (r = -0.195, p ≤ 0.001). Conclusion: This study showed high prevalence of obesity in 8-12-year-old schoolchildren and the association between caries and obesity
Sharma et al. [60]	Evaluate correlation between dental caries (dmft) and obesity (BMI) in 3-6-year-old schoolchildren from urban and rural dwellings.	Cross sectional	1000	India	Outcomes: Male participants and urban residents had significantly higher BMI than female and rural residents respectively (p < 0.05 each). Dental caries was non-significantly different between groups Conclusion: No significant correlation was noted between dental caries and BMI and obesity was more prevalent in urban group when compared to rural group.
Shen et al. [61]	Determine association between severe dental caries [dmft, PUFA (pulpal involvement, ulceration, fistula, and abscess)] and overweight/underweight status (BMI-for-age z-score). For overweight status, BMI cutoff was >2 SD for children under 60 months and >1 SD for over 60 months. For underweight status, BMI cutoff was < -2 SD regardless of age	Longitudinal	772	China	Outcomes: Children in the age range of 24.6-71.1 months were included in the study and median follow up time was approximately 10 months. There was higher odds for underweight status in children with severe dental caries (OR = 4.08). Further, severe caries at baseline had higher odds for overweight status (OR = 2.33). Conclusion: A U-shaped relationship between severe dental caries and both overweight and underweight status was noted.
Swaminathan et al. [62]	Determine correlation between BMI (overweight and underweight) and dental caries (DMFT/DEFT) in 3-12-year-old schoolchildren (2 subgroups by age: 3-5; 6-12).	Cross sectional	2200	India	Outcomes: No significant differences were found for dental caries between overweight and underweight groups (by BMI) Conclusion: No association between BMI and dental caries was found in children in both primary and mixed dentition stages.
Tschammler et al. [63]	Evaluate prevalence and severity of erosive wear (basic erosive wear examination, BEWE) and dental caries (DMFT/dmft, ICDAS) in participants (4-17-year-old) with increased BMI and compare to normal BMI participants. Further, oral hygiene and food consumption habits along with demographic and socioeconomic information was recorded.	Case-control	223 (170 cases, 53 controls)	Germany	Outcomes: Erosive tooth wear and caries burden was significantly higher in obese and extremely obese children when compared to normal weight children (p < 0.05). Increased BMI, older children, male gender, and consumption of erosive foods conferred significantly increased risk for erosive tooth wear and dental caries. Additionally, lower socioeconomic status and poor toothbrushing habits were risk factors for dental caries only. Conclusion: Increased BMI in children and adolescents was significantly associated with increased risk for erosive tooth wear (BEWE) and dental caries (DMFT/dmft, ICDAS)
Metabolic syndrome					
Iwasaki et al. [64]	Evaluate association between metabolic syndrome, diet and dental caries in Japanese adults by comparing participants with and without metabolic syndrome	Cross sectional	937	Japan	Outcomes: For diet, significant differences were noted between participants with and without metabolic syndrome for consumption of dairy products (*p* < 0.01), bean products, sweets and coffee (*p* < 0.05 each); for clinical parameters, significant differences were noted between groups for gender, age, Brinkman index, BMI, waist circumference, blood pressure-both systolic and diastolic, serum fasting blood glucose, high-density lipoproteins (HDL) cholesterol and CRP (*p* < 0.001 each); for oral disease, significant difference was noted between periodontitis and DMFT (*p* < 0.05 each); multivariate logistic regression analysis after adjusting for confounders showed association between metabolic syndrome prevalence and DMFT (first vs. fourth quartile, OR=1.8 and p>0.05) Conclusion: There appears to be positive association between caries and metabolic syndrome in Japanese adults. This association was strong in those with higher DMFT regardless of dietary habits
Adachi et al. [65]	Human prospective study of one-year duration to understand relationship between dental caries, periodontitis, and metabolic syndrome (MetS). Adult participants, ≥ 35 years without MetS underwent assessment by survey, medical and dental examinations and were followed up at one-year interval.	Longitudinal	136	Japan	Outcomes: 30 adult participants demonstrated one or more components for MetS diagnosis. In these participants, DT were significantly associated with development of at least one MetS determining component [RR=3.25 (1.59-6.63)]. No associations between periodontitis and other components of DMFT index were noted. Conclusion: DT may impart an increased risk for subsequent development of MetS.
Animal studies on metabolic diseases and caries					
Diabetes					
Abbassy et al. [20]	Animal study to evaluate morphological and mineral content change of teeth by comparing observations from experimentally induced T1DM and control rats (n=10 each)				Outcomes: T1DM rats showed significant decrease in early weight (day 14 onwards) compared to controls (*p* < 0.05); microtomography of the mandible showed significant reduction in enamel and dentin thickness (*p* < 0.05) when compared to controls; histomorphometry showed significant decrease in mineral apposition and dentin formation rates (*p* < 0.05) when compared to controls Conclusion: T1DM has detrimental influence on the formation of enamel and dentin in early growth stage in a diabetes rat model
Claudino et al. [21]	Animal study to evaluate influence of uncontrolled diabetes on loss of tooth structure by comparing diabetic rat model with controls (n=25 each) over a one-year period				Outcomes: Significantly increased loss of tooth structure was noted in diabetic group at all observation intervals (3,6,9,12 months) when compared to controls; morphometric evaluation of dental pulp showed significant reduction in volumetric density of collagen fibers and fibroblasts when compared to controls (*p* < 0.05) as early as 3 months; non-significant differences in other histological and radiographic criteria were noted between groups, including inflammatory cell infiltrate, necrosis, other connective tissue changes and periapical lesions Conclusion: Uncontrolled diabetes possibly triggers loss of tooth structure and progressive changes of the dental pulp. Therefore, diabetes may be considered a risk factor for development of dental caries and alterations of dental pulp
Nakahara et al. [22]	Animal study to evaluate if hyperglycemia induces periodontal inflammation by comparing results between T1DM diabetic rats (n=30, 10 each in 3 groups) and non-diabetic rats (n=30, 10 in each group) administered variable concentrations of fluoride. Also, a T2DM mouse model (n=30, 10 in each group) was compared with non-diabetic mice (n=30, 10 in each group).				Outcomes: In T1DM rat model, fluoride treatment significantly reduced dental caries, gingivitis and marginal periodontitis in 10 and 50ppm fluoride groups when compared to no fluoride group (*p* < 0.01); the T2DM mouse model, similar results were noted; in fluoride-untreated diabetic rats and mice, marginal periodontitis was always accompanied by moderate caries and alveolar bone resorption or marginal periodontitis was not noted in the absence of caries, regardless of diabetic status Conclusion: Long-term hyperglycemia induces dental caries but not periodontal disease in type 1 and 2 diabetic rodent models
Nakahara et al. [23]	Animal study to evaluate dental caries and periodontal disease in an alloxan induced hyperglycemia (T1DM) rat model (n=30) compared with non-diabetic group (n=17)				Outcomes: Caries score was worse in diabetic group when compared to non-diabetic group; caries severity worsened with age with significantly higher scores at 26 weeks for maxillary (*p* < 0.05) and mandibular (*p* < 0.01) teeth when compared to 13 weeks; mean caries score was significantly higher in mandibular molars when compared to maxillary molars at 13 and 26 weeks (*p* < 0.05 and *p* < 0.01 respectively); alveolar bone resorption was significantly higher in mandible compared to maxilla after 26 weeks (*p* < 0.01) in diabetic group and alveolar bone resorption was noted adjacent to carious molars only; positive correlation was noted between alveolar bone resorption and caries score for both maxilla and mandible (*p* < 0.01 each) Conclusion: Alloxan-induced severe hyperglycemia can cause rapid and progressive dental caries and periodontitis in diabetic rat models
Nakahara et al. [24]	Animal study (n=60, 15 in each group) to evaluate relationship between hyperglycemia and dental caries and preventive effect of glycemic control on progression of caries in diabetic rat model by comparing four groups [spontaneous diabetes-with (INS) and without (C) insulin intervention, alloxan induced diabetes with (AL+INS) and without insulin intervention (AL)]				Outcomes: Mean caries score and mean bone resorption of the maxilla were significantly lower (*p* < 0.01 each) in the INS group when compared to C group; mean caries score and mean bone resorption were significantly lower (*p* < 0.01 each) in the AL+INS group when compared to AL group; positive correlation was noted between alveolar bone resorption and caries scores (*p* < 0.01 for both maxilla and mandible); incidence of marginal periodontitis was significantly lower in INS and AL+INS groups when compared to C and AL groups (*p* < 0.05 and *p* < 0.01 respectively); no periodontal lesions were noted adjacent to a non-carious molar and non-carious molars were comparable in C and INS groups Conclusion: Glycemic control by insulin prevented occurrence and progression of dental caries and caries-related periodontitis in spontaneously and alloxan induced rodent diabetes model, suggesting that hyperglycemia may be a major factor influencing the development of dental caries
Nishimoto et al. [25]	Animal study (n=23, n=13 in diabetic group and n=10 in non-diabetic group) to evaluate relationship between hyperglycemia and early dental caries (7 weeks after alloxan administration) and the role of saliva and salivary glands in a rat diabetes model				Outcomes: Mean weight of saliva (after pilocarpine administration) was significantly lower in diabetic group when compared to non-diabetic group at all time points of sample collection (*p* < 0.01); mean cusp height of molars (a measure of wear) in the diabetic group was approximately half that of non-diabetic group and that difference was significant (*p* < 0.01) as was the incidence and severity of caries; the incidence and severity of histologic change in diabetic group was significantly more than non-diabetic group (*p* < 0.01) and this change was predominantly in terms of vacuolation of acinar cells Conclusion: In this rodent diabetic model, hyperglycemia induces initial caries development and enhances occlusal wear. Also, parotid gland dysfunction may be involved in pathogenesis of occlusal wear and caries
Sano et al. [26]	Animal study to determine if diabetes affects onset and progression of dental caries and periodontal disease over long follow up (20 to 50 weeks). Also, clinical presentation of caries and periodontal disease in a rodent diabetic model were studied by comparing diabetic and non-diabetic mice				Outcomes: Significantly higher incidence and severity of dental caries was noted in diabetic mice at 30 to 50 weeks of follow ups when compared to non-diabetic mice (*p* < 0.05 at 30 and 40 weeks and *p* < 0.001 at 50 weeks); mean caries score was significantly higher (*p* < 0.001) in diabetic mice when compared to non-diabetic mice; severity of gingivitis was positively correlated with severity of molar caries (*p* < 0.001 for both genders and both jaws) Conclusion: There is a strong relationship between diabetes and dental caries in this rodent diabetes model. It is possible that onset of periodontal disease was secondary to dental caries
Yeh et al. [27]	Animal study to elucidate mechanisms of dental caries by studying type 1 diabetic mice model with point mutation in *Ins2 *gene and clinically demonstrates hyperglycemia and xerostomia. The disease model mice were compared with wild-type littermates				Outcomes: Mouse model for type 1 diabetes showed progressive changes in tooth appearance and wear when compared to wild type mice. No differences were noted in tooth development, suggesting these changes occurred due to hyperglycemia and xerostomia. In salivary glands, saliva production was little to none in type 1 diabetic mice after pilocarpine stimulation demonstrating significant xerostomia. Conclusion: Hyperglycemia and xerostomia in type 1 diabetes mouse model leads to excessive dental wear and demineralization.

**Table 3:** Evidence on respiratory diseases and caries

Study	Objectives and study design	Study type	Number of participants	Location of study	Outcomes and conclusions
Human studies on respiratory diseases and caries					
Cystic fibrosis (CF)					
Alkhateeb et al [66]	Evaluate association of unstimulated salivary flow, pH and buffering capacity to dental caries prevalence in CF patients	Cross sectional	83	United States	Outcomes: No significant interaction between unstimulated salivary flow, pH and buffering capacity to dental caries prevalence Conclusion: Future studies should measure other potential biomarkers in saliva of CF patients
Peker et al [67]	Evaluate association of treatment, diet, oral hygiene and salivary factors to dental caries and molar-incisor hypomineralization (MIH) in CF patients	Case-control	60 (30 cases and controls)	Turkey	Outcomes: DMF-T score was significantly lower in CF patients when compared to controls (*p* = 0.001). All other parameters were non-significantly different and 43% of children with MIH used antibiotics Conclusion: MIH frequency and lower caries experience in CF pediatric patients could be related to certain salivary factors or pharmacological therapy
Asthma					
Botelho et al [68]	Evaluate caries risk by studying biofilm control and microbiological factors in addition to caries incidence and severity (DMFT) in a pediatric participants with and without asthma	Case-control	160 (80 cases and controls)	Brazil	Outcomes: No statistically significant difference was noted for caries experience between the two groups. However, in asthma group, significantly higher plaque index and *S. mutans*, positive correlation between *S. mutans* and plaque index and positive correlation between *S. mutans* and duration of treatment were noted Conclusion: Asthma may be a risk factor for increased caries prevalence due to higher *S. mutans* and biofilm accumulation
Cherkasov et al [69]	Compare oral microbiota using 16 S sequencing in pediatric participants with asthma (with and without caries)	Case-control	18 (10 cases, 8 controls)	Russia	Outcomes: No significant differences in oral microbiotas were noted between the two groups. Genus Veillonella was significantly higher in abundance in asthma with caries group and genus Neisseria was significantly higher in asthma without caries group (*p* < 0.05) Conclusion: Veillonella may be related to caries in asthmatic children; potential respiratory pathogens were present in both groups
Ergöz et al [18]	Compare caries experience (DMFT/dmft and DMFS/dmfs) between asthmatic and healt hy children and evaluate genetic association with enamel development genes	Case-control	200 (100 cases and controls)	Turkey	Outcomes: Association between a SNP variation in Ameloblastin gene (*AMBN* rs4694075) and caries experience was noted in asthmatic children (*p* = 2.525e-007) after controlling for confounding factors Conclusion: Ameloblastin is associated with caries in asthmatic children
Heidari et al [70]	Evaluate associations of asthma medications to caries prevalence (DMFT/dmft and DMFS/dmfs) in pediatric cohort	Cross sectional	85	Iran	Outcomes: Significant correlation was noted between tablet form of asthma medications (cetirizine and ketotifen) and DMFT/dmft scores (*p* = 0.006) but no correlations were noted between combination of medications, duration of treatment, quantity of medications used, route of administration and caries prevalence Conclusion: Tablet form of medication significantly increased the severity of dental caries
Stensson et al [71]	Investigate caries determinants in a pediatric cohort with asthma examined at 3 and 6 years and compared with healthy controls	Case-control	114 (64 cases, 50 controls)	Sweden	Outcomes: Significantly higher caries increments in asthmatic children between 3 and 6 years (*p* < 0.05); at 3 years, asthmatic children had significantly higher consumption of sugary drinks and gingival inflammation (*p* < 0.05) and at 6 years, significantly more children were mouth breathers in the asthma group Conclusion: Asthma, intake of sugary drinks more than once daily and increased caries prevalence at 3 years were strongest predictors of developing more carious lesions till 6 years of age
Stensson et al. [72]	Compare caries (DFS) prevalence, dental caries related factors (dietary and oral hygiene habits, cariogenic bacterial counts, and salivary flow and pH) and Cariogram in 12-16-year-old participants with long-term asthma	Case-control	40 (20 cases and controls)	Sweden	Outcomes: Significantly lower salivary flow rate (*p* < 0.05), salivary pH (*p* < 0.05) and significantly higher DFS score (p < 0.01) were noted in the asthmatic group when compared to controls. Cariogram data showed that 10% of asthmatics and 55% of controls had high chance of avoiding caries, a significant difference (*p* < 0.01) Conclusion: Adolescents with long-term asthma showed comparatively higher total DFS and caries risk but reduced salivary flow rate
Hassanpour et al. [73]	Compare dental caries prevalence in asthmatic children on inhaled corticosteroids and healthy children in 3-12-year-old age group. Dental caries was assessed and recorded using DMFT index and information on duration of corticosteroid use was collected	Case-control	140 (70 cases and controls)	Iran	Outcomes: DT (*p* = 0.001) and DMFT (*p* = 0.002)/dmft (*p* = 0.001) were significantly higher in children with asthma that used corticosteroids for 2 years Conclusion: Asthmatic children on inhaled corticosteroids may be at increased risk for dental caries and may benefit from preventive dental programs
Khalifa et al. [74]	Compare dental caries burden (DMFS/DMFT) between controlled asthmatics and healthy controls (age-matched and first-degree relatives). Salivary electrolytes, pH and cariogenic bacterial counts were recorded. Further, duration of asthma and type of medications used were collected (β_2_ agonists with or without corticosteroids)	Case-control	120 (60 cases and controls)	Saudi Arabia	Outcomes: Caries prevalence was higher in asthmatics compared to controls (48.3 and 23.3% respectively). There was a positive correlation between duration of asthma and caries. Cariogenic bacterial counts (*S. mutans, Lactobacilli*) and salivary electrolytes (Ca, K, P) were higher in asthmatics Conclusion: Higher caries prevalence was noted in asthmatics and positive correlation was noted with reduced salivary pH, increased *S.mutans* and *Lactobacilli.*
Animal study on respiratory diseases and caries					
Cystic fibrosis (CF)					
Catalán et al [75]	Animal study to measure effect of *S. mutans* oral inoculation and high sucrose diet by comparing carious lesions in CF and wildtype mice				Outcomes: Significantly higher incidence of carious lesions in CF mice (*p* < 0.003, *t test*); Salivary bicarbonate concentration was significantly reduced in CF mice (*p* < 0.01, *t test*) Conclusion: Decrease in salivary bicarbonate concentration may be partially responsible for increased severity of carious lesions in CF mice

**Table 4:** Evidence on gastrointestinal diseases and caries

Study	Objectives and study design	Study type	Number of participants	Location of study	Outcomes and conclusions
Human studies on gastrointestinal diseases and caries					
Inflammatory bowel disease (IBD)					
Koutsochristou et al. [76]	Evaluate dental caries (DMFT/dmft) and periodontal disease experience [gingival, plaque and community periodontal index of treatment needs (CPITN) indices] in pediatric and adolescent IBD patients and compared with controls	Case-control	110 (55 cases and controls)	Greece	Outcomes: IBD group showed significantly higher DMFT/dmft, gingival inflammation and CPITN index (*p* < 0.001) with non-significant differences in biofilm and oral hygiene habits compared to controls Conclusions: IBD patients under remission showed significantly higher prevalence of dental caries and gingival inflammation despite similar oral hygiene status
Zhang et al. [77]	Evaluate prevalence, severity, and extent of dental caries (DMFT/DMFS) in IBD patients and compare them with healthy controls. Further, questionnaire on demographics, education, smoking, oral hygiene habits, duration of disease and treatment was used to collect information	Case-control	530 (265 cases and controls)	China	Outcomes: DMFS was significantly higher in UC and CD patients compared to controls (*p* < 0.001). Patients with CD and UC had significantly higher risk of dental caries compared to controls (OR = 4.27 and 2.21 respectively). No significant difference for dental caries was noted between UC and CD patients Conclusion: IBD patients in this study had higher prevalence, severity and extent of dental caries and are at higher risk for dental caries compared to controls
Crohn’s Disease (CD)					
Szymanska et al [78]	Human case-control study (n = 225, 150 cases: 71 with resective surgery, 79 without surgery and 75 controls) in CD patients to evaluate association with caries prevalence (DMFT/DMFS), salivary parameters, biofilm control and presence of *S. mutans* and *Lactobacilli.*	Case-control	225 (150 cases and 75 controls)	Sweden	Outcomes: CD patients who had undergone resective surgery demonstrated significantly higher DMFS score (*p* = 0.01), significantly higher counts of *S.mutans* (*p* = 0.04) and *Lactobacilli* *p* = 0.01), significantly higher dental biofilm (*p* = 0.001) and consumption of sweetened drinks between meals (*p* = 0.001) compared to controls Conclusion: CD patients who have undergone resective surgery demonstrate significantly increased risk factors for dental caries when compared to controls
Animal study on gastrointestinal diseases and caries					
Ulcerative Colitis (UC)					
Kojima et al [79]	Animal study to investigate effect of *S.mutans* on dextran sodium sulfate (DSS) induced colitis in a mouse model				Outcomes: A serotype *k* strain of *S.mutans* increased severity of colitis in the mouse model, showed evasion of phagocytosis in the peripheral blood (possibly due to variation in surface glucose side chains) and uptake by hepatocytes (potentially mediated by a collagen binding protein); the serotype *k* of *S.mutans* also mediated increase in IFN-γ Conclusion: Serotype *k* of *S.mutans* is a potential risk factor for UC. Virulence factors of interest include presence of collagen binding protein and lack of certain surface glucose side chains

**Table 5:** Evidence on neurological diseases and caries

Study	Objectives and study design	Study type			Number of participants		Location of study	Outcomes and conclusions
Human study on neurological diseases and caries								
Cerebral palsy								
Cardoso et al. [80]	Determine prevalence and risk factors for dental caries and periodontal disease in children and adolescents with cerebral palsy (CP). DMFT/dmft, gingival bleeding index (GBI) and community periodontal index (CPI) were assessed on oral examination. Additionally, type of CP, socioeconomic status, caregiver’s education level and attitudes towards general and oral health were assessed	Cross sectional			80		Brazil	Outcomes: Participants ranging from 2-18-year-old were included. High prevalence of dental caries (59.3 %) with mean DMFT/dmft of 1.71 ± 2.42 / 2.22 ± 3.23 were noted. Further, caregiver’s education level of less than 8 years was found to be associated with dental caries experience using a Poisson Regression model [PR = 1.439 (1.09–1.89)] Conclusion: CP patients demonstrated high caries prevalence and association with fewer education years of caregivers
Attention Deficit Hyperactivity Disorder (ADHD)								
Paszynska et al. [81]	Determine prevalence of obesity/overweight (BMI) and dental caries (ICDAS) in children (under 11 years of age) with attention deficit hyperactivity disorder (ADHD) and compare with non-ADHD control group. Behavioral evaluation and parent interview for food habits was also conducted		Case-control	119 (39 cases and 80 controls)		Poland		Outcomes: ADHD group had significantly higher BMI compared to controls (p = 0.016). ICDAS scores were significantly different in primary (ICDAS 0,1,2,5,6) and permanent (ICDAS 1,3) dentition. Of note, ICDAS scores 5,6 were significantly higher in ADHD group in primary dentition (*p* < 0.001 and *p* = 0.009 respectively). Food habit interview showed significantly higher proportion of ADHD group participants consume sugary foods and drinks Conclusion: Increased intake of sugary foods and drinks in ADHD patients may lead to weight gain and increased dental caries burden

**Table 6:** Evidence on other diseases and caries

Study	Objectives and study design	Study type	Number of participants	Location of study	Outcomes and conclusions
Human studies on other diseases and caries					
Rheumatoid arthritis					
Ayräväinen et al [82]	Evaluate if oral inflammatory conditions are associated with RA and if targeted treatment of RA impacts oral health by comparing three groups-early untreated RA, chronic RA and controls	Case-control	124 (81 cases and 43 controls)	Finland	Outcomes: Patients in early untreated RA (EURA) group, bronchial asthma was significantly more common compared to other groups (*p* = 0.04); during the study, significant decrease in pain in joints in both RA groups (*p* < 0.001) and significant increase in patients who brushed twice a day (*p* = 0.04) occurred; DMFT and DMFS was non-significantly different between groups and total dental index (TDI) was significantly worse in RA patients compared to controls (*p* = 0.04); number of 4–5 mm pockets were significantly higher in EURA group vs. chronic RA patients at both baseline and follow up visits (*p* < 0.001); positive Rheumatoid factor (RF) was associated with oral inflammation and patients with positive RF had higher median TDI values and deep pockets (≥4mm) compared to patients with negative RF (p = 0.001 and *p* > 0.001 respectively); DMFT, DMFS, TDI indices and serologic markers (SSA/SSB and RNP antibodies) were significantly higher in EURA patients compared to other groups (*p* = 0.039); DMFT and DMFS was associated with disease activity score (DAS28) in both RA patient groups at baseline and follow up; DMFS had an increasing association with activity of RA (*p* < 0.001) Conclusion: RA patients had poorer oral health compared to controls and caries indices were associated with RA activity
Martinez-Martinez et al [83]	Evaluate frequency and severity of dental caries and the counts of cariogenic bacteria in RA patients when compared to controls	Case-control	160 (80 cases and controls)	Mexico	Outcomes: RA patients reported significantly higher dry mouth sensation compared to control group (p < 0.05); RA group had significantly higher decayed teeth (p = 0.0149) and lower count of FT (p = 0.0004) compared to control group; treatment needs index and care index were significantly higher in RA and control groups respectively (p = 0.0002 and p = 0.0009 respectively); *S. mutans* was significantly higher in RA patients compared to controls (p < 0.05) Conclusion: Dental caries is more frequent and severe in RA patients
Systemic lupus erythematosus (SLE)					
Loyola Rodriguez et al [84]	Compare the prevalence of dental caries in active and inactive SLE patients by evaluating clinical, salivary and bacterial factors	Case-control	60 (30 cases and controls)	Mexico	Outcomes: DMFT index was significantly higher in active SLE group when compared to inactive SLE group (*p* < 0.05) and only decayed component of DMFT index showed significant difference; functional teeth index (FS-T), treatment needs index (TNI) and care index (CI) were significantly different between groups (*p* < 0.05); integrative dental caries index (IDCI) showed significantly higher caries severity in the active SLE group for mild and moderate degrees of caries severity (*p* < 0.05); salivary flow and pH were significantly reduced in the active SLE group compared to inactive group (p < 0.05); DNA copies of *S. mutans* and *S. sobrinus* were significantly different between groups (p < 0.05); correlation was noted between SLE disease activity and DMFT, caries surfaces, pH, salivary flow, *S. mutans* and *S. sobrinus* and total bacteria (p < 0.0001) Conclusion: SLE patients had high DMFT and active SLE patients had significantly high smooth surface caries along with high counts of *S. mutans* and *S.sobrinus.* A positive correlation was noted between active SLE and dental caries
Gofur et al. [85]	Compare dental caries burden in patients with increasing severity of SLE. DMFT, oral hygiene metrics [oral hygiene index-simplified (OHI-S), debris index (DI), plaque index (PI), calculus index (CI) and personal hygiene performance-modified index (PHP-M)] and SLE severity (SLE disease severity index) were collected	Cross sectional	93	Indonesia	Outcomes: Patients were divided into mild, moderate and severe SLE disease severity. 74% patients with SLE had dental caries. PHP-M (*p* < 0.001), PI (p = 0.001), OHI-S (*p* < 0.001), DMFT (*p* = 0.001) were significantly associated with SLE severity Conclusion: Correlation between oral hygiene metrics, dental caries and SLE severity was found in SLE patients
Chronic kidney disease					
Menezes et al [86]	Evaluate the association between chronic kidney disease and dental caries by comparing dental caries, salivary factors and cariogenic bacteria between patients with end stage renal disease and matched controls	Case-control	214 (107 cases and controls)	Brazil	Outcomes: Significantly higher CFUs of *S. mutans* (*p* = 0.02), amounts of anti-mutans IgA (*p* = 0.04), urea in saliva (*p* < 0.001) and significantly fewer FT (*p* < 0.001) in group with end stage renal disease; presence of *S. mutans*, lower DMFT, fewer FT, lower salivary calcium and higher salivary urea were associated with end stage renal disease; positive correlation between quantity of anti-streptococcus IgA antibodies, salivary urea, colony forming units (CFUs) of *S. mutans* and duration of hemodialysis were noted; negative correlation was noted between hemodialysis and FT Conclusion: Programs to prevent and treat oral problems in end-stage renal disease patients on hemodialysis may be required to improve awareness and oral health condition

## Discussion

### Cardiovascular diseases and caries

Dental caries experience was not significantly different when compared to controls in human studies of coronary and peripheral arterial disease, but overall oral inflammatory burden was significantly higher in cases due to increased burden of periodontal disease [29, 31]. Additionally, one study compared dental caries in patients with congenital heart disease with and without heart transplants, thus comparing the effect of immunocompromised status on dental caries [30]. Interestingly, transplant group had significantly lower caries experience when compared to group without heart transplant. The authors explained this difference based on the possibility of increased attention to dental care in children with heart transplants. While frequent antibiotic intake could explain this observation, in this study it was an exclusion criteria due to possible confounding of results.

Other studies have evaluated attitudes of dentists and of parents of children who are at high risk for infective endocarditis [87] including those with congenital heart disease [88–90]. These findings suggest that specialists in pediatric dentistry and general practitioners who regularly treat children are more informed about appropriate dental care for children with congenital heart disease. There needs to be a concerted effort between the dentists, medical providers and parents to encourage prevention to achieve favorable outcomes in children at high risk for infective endocarditis due to their cardiac conditions.

More atherosclerotic plaque and presence of genomic DNA from *S.mutans* was found in a group of ApoE^null^ mice, infected by intravenous injection (tail vein) of *S.mutans* and subjected to balloon angioplasty injury compared to non-injured mice (controls) [33]. Immunohistochemically, sections of atherosclerotic plaque from injured group showed macrophage invasion in the tunica adventitia of aorta and upregulation of TLR4. Further studies demonstrated that collagen-binding protein (*cnm)* is important for invasive potential of *S .mutans* [13]. Specifically, it was found that serotype *f* strain OMZ175 of *S. mutans* has this capability [91]. These studies further explores the invasive nature of serotype *f* strain OMZ175 of *S. mutans* in a model of cellular injury.

While experimental data on the invasive potential for certain serotypes of *S. mutans* exists, expert panels do not recommend antibiotic prophylaxis prior to all dental procedures. The rationale behind such an approach is that the likelihood of developing infective endocarditis due to a bacteremia from dental procedure is significantly lower than bacteremia from routine at-home toothbrushing and flossing. Furthermore, it is not clear if antibiotic prophylaxis prior to dental procedures will prevent all potential for infective endocarditis secondary to dental procedures. In this scenario, the risk-benefit analysis appears to be of low benefit and high risk, taking into account the potential for antibiotic resistance. A recent meta-analysis of randomized controlled trials showed vesicoureteral reflux patients treated with antibiotic prophylaxis were 6.4 times more likely to develop a multidrug-resistant urinary tract infection [92]. It is reasonable to use conclusion from this study and exercise caution in frequent antibiotic prophylaxis for dental procedures till directly applicable results are available in the dental literature. While caution must be exercised, there are exceptions and it is thought that patients in certain high-risk categories may benefit from antibiotic prophylaxis [93].

Ostalska-Nowicka et al. found association of dental caries with primary hypertension in a case-control study [32]. Authors found significantly higher salivary evening cortisol levels, uric acid concentrations in participants with caries and also found correlation between dental caries and microalbuminuria. These biochemical parameters are of importance in pathophysiology of hypertension and indicative of activation of renin-angiotensin system and reorganization of endothelium [32]. Considering the multifactorial nature of dental caries and hypertension, future studies that evaluate social determinants, diet and systemic inflammation secondary to oral and gastrointestinal dysbiosis may provide valuable input into common mechanisms of dental caries and primary hypertension [94–97].

### Metabolic disorders and caries

#### Diabetes

Several human clinical studies and animal studies have addressed the connection between dental caries and diabetes. Outcomes other than caries were also studied, including salivary composition, microbiology and periodontal status. Hegde et al. found that caries active participants who were diabetic demonstrated significantly reduced salivary calcium and significantly increased alkaline phosphatase when compared to caries active non-diabetic participants [34]. Similarly, Al-Badr et al. demonstrated that children with type 1 diabetes had significantly lower salivary pH and higher counts of *Lactobacilli.* Reduced salivary pH and higher lactobacilli count are crucial factors for demineralization of teeth and exacerbation of dental caries [38]. Reduction in salivary pH and increase in counts of cariogenic microbiota can occur secondary to cariogenic diet and poor plaque control and was demonstrated as such by Kamran et al. [39] and therefore, it is important to emphasize the multifactorial and overlapping nature of dental caries and obesity before drawing conclusions from study of select variables. Furthermore, studies into the association of diabetic control and other parameters of diabetes phenotype with dental caries will increase our understanding of risk stratification and consequently, prevention of dental caries in diabetic patients [40, 41]. Two other studies showed that lifestyle, dietary and oral care factors were significantly different between groups with controlled and uncontrolled diabetes measured by glycated Hb [35, 36]. Similarly, when pediatric cohort with phenylketonuria and those with type 1 diabetes were compared, children with phenylketonuria had significantly higher caries experience [37].

Animal studies used rodent models of diabetes (primarily type 1 diabetes) and hyperglycemia to study its relationship with dental caries and other tooth-related changes [20–27]. Changes in enamel, dentin, pulp and salivary glands with alveolar bone loss were compared, both to control groups and groups with intervention using fluoride application and insulin administration. Consistent results from animal models demonstrated that hyperglycemia in diabetic rodents was associated with increased dental caries [22–26]. In addition, these studies showed that there were histological and morphometric changes in enamel, dentin and pulp in diabetic animals. There were reduction in volume of pulpal connective tissue and enamel and dentin, along with excessive wear of enamel [20, 21, 25, 27]. Salivary histological change included vacuolization in acinar cells and functionally, reduction in saliva production that resulted in xerostomia [25, 27]. Carious lesions positively correlated with gingivitis and periodontitis [23, 26]. Lastly, both fluoride application and insulin administration interventions resulted in reduction of dental caries, marginal gingivitis and periodontitis [22, 24].

#### Obesity

Of all systemic diseases, an association between obesity and caries was more robust than noted for other systemic conditions, as documented in twenty-two human clinical studies including eight longitudinal clinical studies. Data from longitudinal studies did not consistently find an association between obesity and dental caries and studies with larger samples sizes did not find association between dental caries and obesity [42, 43, 47, 48, 51–53, 57, 58, 60, 62, 63]. In studies where obesity and metabolic syndrome were found to be associated with caries, odds ratio ranged from 1.01 to 3.7 [42, 43, 49, 52, 63–65]. Interestingly, a relationship between low BMI and dental caries was noted and an inverse relationship between overweight status and caries was seen in some studies [44, 46, 58]. Chala et al., through statistical modeling found a U-shaped relationship between BMI and caries, which means that caries was associated with both underweight and overweight status [44] and this U-shaped relationship between BMI and caries has been reproduced in two recent studies. Untreated dental caries can impact overall nutritional status and subsequently BMI. Further, reduction in masticatory efficiency can promote intake of softer foods and increase in dental caries burden [56, 61]. Longitudinal studies are needed to examine relationship between onset and progression of dental caries and their effect on BMI. A study showed significant weight gain in children when teeth with severe dental caries and pulpal involvement were extracted [98]. Mixed results on the association of BMI and dental caries are also indicative of the complex etiologic nature of dental caries. Various factors including access and attitude to dental care, socio-economic status, maternal education, oral habits, diet, biological and microbiological factors interact in caries etiopathogenesis [99]. Additionally, variable definitions and surrogate markers used in association studies further complicate consensus and ability to synthesize reproducible conclusions [100]. An important implication of the mixed results observed for caries association with systemic conditions likely results in lack of reliable, reproducible risk prediction tools for dental caries [101]. It appears that past and current caries experience along with frequent follow ups and use of fluoride for caries prevention remain the most effective tools for caries prevention in clinical practice.

### Respiratory diseases and caries

#### Asthma

Most human clinical studies were undertaken in pediatric cohorts and were case-control in design, aimed at comparing groups with asthma and caries to groups with caries alone. Caries burden was typically measured using DMFT/dmft and DMFS/dmfs indices along with other variables, including microbiological (*S. mutans* and *Lactobacilli* counts, oral microbiota assessment using 16 S sequencing) [68, 69, 74], medications [70], sugary diet [71], salivary parameters [72, 74] and genetics [18]. Results reinforced previously discovered etiological factors for dental caries in children; namely, consumption of sugary drinks [71], higher *S. mutans* counts, higher plaque index in caries active children [68], and lower salivary flow rate and pH [72, 74]. Other factors related to dental caries activity included tablet delivery of asthma medication [70], the abundance of *Veillonella* sp. [69] and SNPs of the ameloblastin gene (*AMBN* rs4694075) [18].

Heidari et al. explained the association of higher caries burden with asthma to the use of tablet form of asthma medication on grounds that tablet formulation delivers a higher drug dose when compared to syrup and spray forms of medications[70]. It is possible that these patients presented with severe symptoms of asthma and therefore required higher drug dose, but that information was not clearly presented. Other studies have shown an association between inhaled corticosteroids for asthma and higher burden of dental caries [73, 102, 103]. Additionally, while the duration of intake of medication was not associated with severity of caries in this study, there are other studies that found contrary results [68, 104]. Cherkasov et al. found an increased relative abundance of *Veillonella* from dental biofilm in caries-affected children when compared to caries-free children with asthma [69]. While *Veillonella* is not considered a cause of dental caries like *S. mutans*, these results are not surprising. *Veillonella* can metabolize lactate which is produced in abundance by cariogenic streptococci in dental biofilms [105], and various studies have previously demonstrated increased levels of *Veillonella* in carious lesions [106–110].

Ergöz et al. found an association between *AMBN* rs4694075 and dental caries in asthmatics, which is an interesting finding. It should be noted that other genome wide association studies (GWAS) studies and GWAS meta-analyses arrived at differing conclusions [111, 112]. Additionally, Ergöz et al. did not mention any dental developmental defects in their cases and so the potential of these being confounding factors may not be applicable. However, it may be argued that since mutations in ameloblastin (*AMBN*) and other dental development genes are related to dental developmental defects [113], clinical information on absence of dental developmental defects may be considered when evaluating and reporting genetic association of dental development genes and asthma.

While it appears that nature of the association of asthma with dental caries is uncertain, it is prudent to employ prevention strategies for dental caries in asthma patients [114, 115].

#### Cystic fibrosis (CF)

Two human studies described an association between dental caries and CF [66, 67]. These studies evaluated caries, molar-incisor hypomineralization, oral hygiene, diet and salivary factors in groups with and without CF. Peker et al. noted lower caries experience (DMF-T) in CF patients, and suggested this could be related to frequent use of antibiotics [67]. Salivary factors studied were not significantly different between the groups. Contrary to human clinical studies, a CF mouse model showed significantly higher caries experience and significantly reduced salivary bicarbonate concentration in CF mice [75]. Although human salivary studies of CF did not show significant differences between controls and cases, interest in exploring salivary biochemical composition in CF patients is practical. CF is caused by mutations in the CFTR gene (CF transmembrane conductance regulator) which is a chloride and bicarbonate channel [116] and CFTR mRNA has been localized in the ductal cells of salivary glands [117]. In a mouse model with deletion of phenylalanine 508, significantly increased counts of *S.mutans* along with increased caries incidence and severity were noted. In the same mouse model, salivary bicarbonate concentration was significantly reduced when compared to wildtype littermates [75]. However, human studies on salivary parameters in CF patients do not consistently show low pH and higher caries severity and systematic review on this data has shown limitations in study design and high risk of bias [118].

### Gastrointestinal diseases and caries

Limited clinical studies were found assessing a connection between caries and gastrointestinal diseases, including studies of inflammatory gastrointestinal diseases [76–78]. A human clinical study (case-control) showed that pediatric participants with inflammatory bowel disease had significantly more caries and periodontal inflammation than healthy participants [76, 77]. Similar results in an adult cohort has been shown previously [119]. In another study, CD patients who had resective surgery demonstrated a greater caries experience, cariogenic microbiota, oral hygiene and poor diet when compared to controls [78]. In other studies, CD patients have demonstrated increased caries prevalence [77, 119, 120], increased sugar intake [121, 122] and increased levels of *S. mutans* [123], one of the bacterial species strongly linked to caries activity. The presence of these factors creates a conducive environment for accelerated caries activity. Patients with IBD have shown increased odds for dental caries in a recent study, confirming previous results in aforementioned studies (4.27 for CD and 2.21 for UC) [77].

Kojima et al. undertook an interesting study using a colitis mouse model to study effects of a serotype of *S. mutans.* Results showed that serotype *k* of *S. mutans* was able to evade host response in peripheral blood due to variation in glucose surface side chains. Also, uptake of *S. mutans* by hepatocytes, which was potentially facilitated by collagen binding protein, aggravated colitis due to production of IFN-γ by liver [79].

*S. mutans* can be divided into four serotypes (*c, e, f, k*) and serotypes *f* and *k* predominantly carry the *cnm* gene. The presence of *cnm* gene confers a collagen binding property to specific *S. mutans* serotypes and has been demonstrated to be essential for invasiveness into human coronary artery endothelium [13]. In the study by Kojima et al., significantly more IBD patients showed *cnm* encoding *S. mutans* (serotype *k* or *f*). Of note, this study showed hepatocyte involvement by *S. mutans* as a crucial step in the colitis mouse model while *S. mutans* was undetectable in samples from the gut [79]. This observation suggests that oral microbiota may affect a disease state in an organ that it does not invade directly by modulating the inflammatory environment. These authors have published follow up studies, investigating the relationship between *S. mutans* serotype *k* and liver disease in mouse models. These studies showed aggravation of non-alcoholic steatohepatitis by a specific strain of *S.mutans* through participation of cell surface proteins, including collagen-binding protein [124, 125].

### Neurologic diseases and caries

Cardoso et al. studied dental caries burden in patients with cerebral palsy and noted that these patients had high prevalence of dental caries and mean DMFT/dmft values of 1.71 and 2.22 respectively. Further, they found that caregiver awareness and education was associated with dental caries experience in these patients (PR = 1.439) [80]. Control of dental biofilm in patients who are limited in their physical and mental capability is challenging [126, 127]and becomes the collective undertaking of caregiver and dentist and thus, education of the caregiver plays a crucial role in achieving this collective goal [80]. Similarly, enhanced preventive measures can be extended in the geriatric population where compromised motor control and masticatory efficiency results in a shift to a softer diet and that along with exposed root surfaces further increases possibility of dental caries [128, 129].

Delwel et al. raised an important point about composite nature of DMFT index, wherein caries experience (decayed component of DMFT) should be evaluated separately to assess caries burden and for statistical comparison between groups [130]. Overall, standardization in dental indices and utilization of semantics and ontology frameworks should enhance our ability for data analysis and draw robust conclusions.

In a case-control study of participants with ADHD, Paszynska et al. found significantly higher BMI in test group and significantly higher ICDAS 5 and 6 scores (teeth with advanced caries) in the primary dentition. They also found that increased intake of sugary foods and drinks were significantly higher in ADHD group[81]. It appears that in studies determining associations of dental caries with neurological and behavioral disorders, standardized interviews for caretakers, food habits and other social determinants will be crucial in order to draw informed conclusions and not extrapolate mechanistic links between systemic diseases and dental caries.

### Other diseases and caries

In our review of literature, we found a few articles that could not be categorized by organ system and/or were not adequate in number to warrant their own section in this review. These diseases included RA, chronic renal diseases and systemic lupus erythematosus.

DMFT/DMFS were associated with RA as assessed by disease activity score and serologic markers. Also, *S.mutans* was significantly higher in RA patients and indices for oral inflammatory burden and disease were associated with serologic markers for RA. Furthermore, the oral inflammatory burden was significantly higher in early untreated RA when compared to chronic RA [82]. RA is a risk factor for both caries and periodontal disease [3]. The plausible link between compromised plaque control and joint dysfunction is reasonable but the contribution of RA to overall inflammatory burden is also an important consideration [131]. Active SLE patients showed increased dental caries activity including smooth surface caries when compared to inactive SLE. Furthermore, salivary pH and flow were significantly reduced and high counts of *S.mutans* were noted [84]. Another study showed relationship between compromised biofilm control, SLE severity and dental caries [85]. These results raise the possibility of SLE being coincidental in the direct relationship between poor oral hygiene and dental caries. In one study that explored associations between dental caries and chronic kidney disease (CKD), disease group demonstrated significantly higher CFUs of *S.mutans* and IgA response but significantly lower filled teeth when compared to controls [86]. Importantly, in this study, plaque index was similar between the two groups. Other studies have demonstrated lower dental caries in CKD patients [132, 133] and proposed the need for longitudinal studies exploring association between dental caries and CKD.

Although evidence of the association between SLE, RA and dental caries is limited, risk stratification of patients in consultation with rheumatologist may facilitate preventive dental care. Determination of xerostomia in patients with SLE or RA is advised since these patients often have associated Sjögren's syndrome (secondary Sjögren's syndrome) and may experience higher dental caries burden secondary to xerostomia, requiring preventive measures [134]. Sjögren's syndrome is a chronic inflammatory autoimmune disease that usually involves exocrine glands including salivary glands [135]. Additionally, other risk factors such as past caries experience, ability to maintain oral hygiene if limited by joint dysfunction and root exposure commonly noted in elderly patients may help with development of a customized dental caries preventive care plan. Similarly, diet in patients with CKD tend to be carbohydrate rich and that along with poor oral hygiene can increase risk for dental caries [132]. In the caries prevention plan for patients with CKD, oral hygiene should be maintained meticulously through proper home care and periodic dental appointments.

The oral cavity has evolved with a symbiotic and diverse microbiota which serves under some circumstances as a safeguard against numerous environmental challenges [136]. Conditions that disrupt this balance include breach in mucosal defenses and acquisition of pathogenic species, or pathogenic traits by certain commensal microbiota. Examples of acute local infections that occur secondary to breach of mucosal barriers and/or colonization by pathogenic microbiota include dental abscess and lymphadenopathy. Dental caries may also influence a systemic response through direct extension of pertinent microbiota or resulting inflammation. A systemic exposure to effects of caries is also plausible through marginal caries that extends to the periodontium or by pulpal involvement. The systemic influence of dental caries both by direct extension of oral microbiota and creation of a pro-inflammatory state are reasonable hypotheses. However, the obvious challenge is to prove such hypothetical mechanisms by human studies. The chronicity of caries as a disease and the ethical challenges imposed by not treating dental caries are significant challenges for future studies.

We expect that mechanistic explanations of dental caries-systemic disease associations in future studies will likely come from animal models. Also, animal models can inform human studies on variables of interest. In this regard, longitudinal studies that are aimed at evaluating oral and systemic variables of interest in periods of accelerated dental caries in the human host will prove useful. This study is a scoping review and provides an overview of available evidence on the topic of associations between dental caries and systemic diseases. It has a wider scope and does not limit the analysis to one systemic disease. This analysis is not as rigorous as that offered by systematic reviews and meta-analyses and the information presented should be used in complement to systematic reviews and meta-analyses on this topic.

## Conclusions

Limited clinical evidence was found connecting several systemic diseases and dental caries. When adequate clinical results were available, it offered mixed evidence of such associations. Interesting animal studies were noted that could generate clinical hypotheses and further investigations in rodent models for cardiovascular injury and hyperglycemia. Best evidence from human and animal studies described the association between metabolic diseases and dental caries. Animal studies using rodent models demonstrated significant changes in dental tissues following hyperglycemia. Also, an association between hyperglycemia and dental caries was consistently noted in animal studies. Inadequate data was found to suggest any modifications to current clinical practice or prevention guidelines.

## Data Availability

Not applicable.

## Abbreviations

AD: Alzheimer Disease
ADHD: Attention Deficit Hyperactivity Disorder
BEWE: Basic Erosive Wear Examination
BMI: Body Mass Index
CAMBRA: Caries Management by Risk Assessment
CKD: Chronic Kidney Disease
CFU: Colony Forming Unit
CPITN: Community Periodontal Index of Treatment Needs
CAD: Coronary Artery Disease
CRP: C-Reactive Protein
CD: Crohn’s Disease
CF: Cystic Fibrosis
CFTR: Cystic Fibrosis Transmembrane Conductance Regulator
DI: Debris Index
DT: Decayed Teeth
DFSa: Decayed, and Filled Approximal Surfaces
DEFS: Decayed, Extracted, and Filled Surfaces
DMFS/dmfs: Decayed, Missing, and Filled Surfaces
DMFT/dmft: Decayed, missing, and Filled Teeth
DSS: Dextran Sodium Sulfate
T1DM: Diabetes Mellitus Type I
T2DM: Diabetes Mellitus Type II
FT: Filled Teeth
GWAS: Genome Wide Association Studies
GUDC: Grade of Severity of Untreated Dental Caries
HDL: High Density Lipoprotein
IBD: Inflammatory Bowel Disease
ICDAS: International Caries Detection and Assessment System
IOTF: International Obesity Task Force
MeSH: Medical Subject Heading
MetS: Metabolic Syndrome
MT: Missing Teeth
MIH: Molar-Incisor Hypomineralization
OR: Odds Ratio
OHI-S: Oral Hygiene Index-Simplified
PHP-M: Personal Hygiene Performance-Modified Index
PI: Plaque Index
RA: Rheumatoid Arthritis
S-ECC: Severe Early Childhood Caries
SiC: Significant Caries Index
SLE: Systemic Lupus Erythematosus
TLR: Toll-like Receptor
TDI: Total Dental Index
UC: Ulcerative Colitis
WHO: World Health Organization
